# Financialization, religion, and social trust in rural China

**DOI:** 10.1371/journal.pone.0240114

**Published:** 2020-10-12

**Authors:** Wei Yin, Berna Kirkulak-Uludag, Kent Matthews

**Affiliations:** 1 School of Economics and Management, Southeast University, Nanjing, China; 2 Faculty of Business, Dokuz Eylul University, İzmir, Turkey; 3 Cardiff Business School, Cardiff University, Cardiff, United Kingdom; 4 Nottingham University Business School Ningbo, Ningbo, China; 5 School of Public Finance & Taxation, Zhongnan University of Economics & Law, Wuhan, China; Technical University of Kosice, SLOVAKIA

## Abstract

This paper examines the impact of financial development and religion on social trust in rural China. We use multinomial logistic regression models with the Chinese Household Income Project (CHIP) Survey Data of 2013. The findings show that while financial development has a negative and significant impact on *particular trust* but no impact on general trust, religion has a positive relationship with general trust but insignificant relationship with particular trust. This study further investigates the impact of interaction between financial development and religion on social trust. The joint effect of financial development and religion has significant and positive relationship with *particular trust*. This implies that while financialization destroys the traditional relatives and friends trust based on lending and borrowing in rural area, religiosity lessens the negative impact of financialization on *particular trust*.

## Introduction

Social trust can be simply described as a belief in the honesty, integrity, and belief in community that engenders cooperation [1]. In its absence, “the alternative” is mistrust fuelled by uncertainty. However, the modern picture is more complex. It can be argued that in a fast-evolving world of globalization, mass-migration, technological developments, environmental and public health threats, building, restoring, and sustaining social trust is harder than ever.

Arguably, economic growth and financial development is associated with improved welfare. However, this is a questionable view. Can financial development be the solution to the spread of social peace and trust in society? To answer this question, one should have a more comprehensive understanding of what financial development means to people and its link with social trust.

Financial development is a broad term, which describes the degree to which the financial sector is developed, but popularly the term ‘financialization’ describes an increasing share of financial services in the economy. The concept also refers to the strength and stability of financial institutions including the legal and regulatory environment [2, 3]. Above all, financial development also covers the depth and access to information, capital, and other financial services. Simply put, households and firms can consume and invest due to financial development. We use the terms financialization and financial development interchangeably.

The link between religion and social trust has also been an avenue of extensive research [4–6]. While differences in trust and religious denominations vary, one strand of the literature finds that religion fosters social trust. Religiosity contributes to the shaping of individual behaviour. In general, religions direct people to behave morally. Here, we aim to explore the impact of financial development (financialization) and religion on social trust in rural China. The implicit link between religion and finance in China is not new. The role of religion in the fostering of trust in enabling the use of trade credit was studied by [7]. They find that firms located in areas of high religiosity are associated with more trade credit. Since trade credit is normally uncollateralized, this transforms into an issue of trust. Significant though this study is, the triangular relationship between financialization, religiosity, and social trust has not been widely studied in China.

The extant literature examines the impact of social trust on financialization and concludes that social trust plays a significant role in the degree of financial development [8–10]. Interestingly, there have been as yet no studies investigating the opposite effect—the impact of financialization on social trust. However, as social trust affects financialization significantly, the causation may be reversed because usually along with financialization, emerges strong financial institutions, effective implementation of financial reforms, investor protection laws, and contractual arrangements [11]. Financialization may encourage people to use formal tools and lessen the need for informal loans from their friends or kinship networks. In financially developed economies, people may rely more on others and feel secure as a result of prompt legal enforcement.

Since the implementation of reforms and open-door policy in 1979, China has experienced remarkable financialization, the impact of which on social trust remains unexplored. The Chinese government made significant progress in establishing financial institutions and growing financial assets over four decades. Financial restructuring gained momentum particularly in late 1990s prior to China’s accession to WTO. In recent years, China’s banking industry has become the largest. Similarly, stock and bond markets are, respectively, ranked second and third in the world in terms of market capitalization [12]. In the wake of China’s financialization, the Chinese government has put considerable effort on increasing access to financial services particularly in rural areas. The consolidation and commercialization of urban and rural cooperatives; creation of the Postal Savings Bank of China; the relaxing of market-entry requirements for rural banking; expansion of formal and informal financial service providers, and intensive investment in FinTech have contributed to financialization in rural China.

In the past three decades, China has experienced both financialization and a rise of religiosity. This has partly arisen out of a relaxation of government religious policies [13], and greater tolerance of religion as a private experience that does not threaten collective authority [14]. The rise of religiosity is strongly associated with China’s economic boom and rapid modernization. The economic rise of China accompanied with rapid urbanization and the sharp shift from a planned to a more market-based economy has brought substantial benefits to the society. However, the benefits came with several social and economic problems associated with rural-to-urban migration, unemployment, involuntarily retirement, and increased marital breakdown; inducing a spiritual hollowness that was arguably filled with religion [15, 16].

Rural residents develop their own social relationship based on family networks within a narrow range. Unlike urban China, where there is greater policing of laws and regulations, rural China tends to rely more on moral standards governed by religious precept [17]. It is also true that religious involvement may help rural society to break the limitation of a kinship network, providing new networks and social ties [18].

The success of the Chinese economy, financialization, and religious freedom is intertwined with the role of the Chinese Communist Party (CCP). But, it was the same CCP, that implemented repressive religious policies following the Cultural Revolution, and initiated the policy of religious tolerance following the the opening-up policy. Having an atheist ideology, the CCP has more than 90 million members all over China. Most members are among relatively well educated and middle-income urban residents who are ambitious to climb the career ladder [19]. In this context, a more nuanced understanding of the determinants of social trust requires also the study of the role of religion and CCP membership in promoting social trust in China.

Using the CHIP survey data of 2013, we examine the impact of financial development (financialization) on social trust in rural China. In order to have a deeper understanding of social trust we divide trust into particular trust and general trust based on previous studies of [20–23], Particular trust is observed when people trust specific people including a fairly small circle such as friends, family members or neighbours. Particular trust is influential in small, face-to-face communities where people interact closely [24–26]. General trust applies to a wider circle of individuals who are acquaintances or strangers. General trust is functional for interactions between unfamiliar people [27–29].

The interaction of financialization and religiosity in rural China remains a relatively unexplored area. While financialization and trust has an ambiguous relation, it is generally felt that religiosity fosters trust. The contribution of this paper is to examine the interaction of financialization and religiosity in its association with trust. Our findings are as follows. First, financialization has a negative impact on *particular trust*, but has an insignificant effect on general trust. Trust within a close social circle is associated with heavy reliance on informal financial settings. Financial development, in rural areas, lessens or eliminates the need for financing from the close social circle. We also find that only individuals with a higher level of education can mitigate the negative effects of financial development on *particular trust*. Moreover, the findings suggest that religion has no significant impact on *particular trust* but has significant and positive impact on general trust. People have a highly developed level of trust with their close circle; however, religion plays the crucial role of enhancing trust. We find that while financialization weakens trust, religion combined with financialization mitigates this negative effect and overall areas of high religiosity are associated with higher levels of trust. Surprisingly, being a CCP member has no impact on social trust in rural China but that membership of a village or township cadre does. This result is interesting given that the Chinese have no alternative political party preference. This finding recognises that urban residents are more likely to become CCP members than rural residents due to their ambition in employment choice and earnings.

The remainder of this paper is organized as follows. Section 2 reviews the relevant literature. Section 3 introduces the data and methodology. Section 4 reports the empirical findings and Section 5 provides the conclusion.

### Literature review

Several empirical studies have focused on the sources of social trust. The frontiers of this field provide substantial works on the impact of social capital and trust on economic development [1, 30–32]. These early studies examined how social trust affects economic and financial development. However, the literature later shifted towards providing answers to the question of how economic growth affects social trust [33, 34] and the recent literature has turned to investigate the relationship between financial development and social trust [8–9, 35].

Calderon et al. [8] examined the relationship between social capital and measures of financial development in a cross-section of countries in the period 1980–1995. They document that social trust is strongly associated with a high degree of financial efficiency. Guiso et al. [9] examine the effect of social capital on financial development across different parts of Italy and conclude that the effect of social capital is stronger where legal enforcement is weaker and among less-educated people. Using household survey data of 28 transitional countries, Afandi [35] examines the effect of social trust on the use of banking services. The findings show social trust has a positive impact on the use of banking services. A high level of social trust leads to intensive use of banking such as bank accounts and bank cards. In another study, Delhey and Newton [36] suggest that income inequality erodes social trust among people. Similarly, Barone and Mocetti [37] show that static inequality (measured by the Gini index and top income shares) and dynamic inequality (proxied by intergenerational income mobility) decreases the level of social trust in society. Using panel data from 82 countries between 1973 and 2008, Elkhuizen et al. [10] find evidence that the success of financial liberalization in promoting financial development is conditional on the prevailing level of social capital.

A further strand of the literature has examined the relationship between religion and social trust with inconclusive results. While some researchers posit that religion has a negative effect on social trust [2, 33, 38, 39], others assert that religion plays a crucial role as a source of networking, and has a positive effect on social trust [36, 40, 41]. The heterogeneous effect of different religions has been reported by Stulz and Williamson [42] who find that Catholic countries have significantly negative relationship with creditor rights protection. Other studies show that religion has no impact on social trust. In their study, Welch et al. [43] use National Election Study data in US and analyze the complicated effects of religions on social trust. The main finding of their study reveals that even the most conservative traditions do not reduce social trust levels. Olson and Li [44] use the World Values Survey data from 77,409 individuals in 69 countries and argue that neither religious concentration nor religious heterogeneity has a significant relationship with social trust.

Another strand of the literature analyses the relationship between membership of voluntary associations and social trust [45–47]. Previous studies provide evidence that associations have high likelihood to promote social trust among their members through norms and regulations [48, 49] or reciprocity from in-groupers [50]. However, some studies fail to find strong evidence of high levels of social trust among the supporters of political parties. For example, using European Social Survey for Nordic countries, Koivula et al. [51] find that the trust level among the supporters from populist parties is comparatively low.

In China, the negative impact of inequality on social has been examined by several researchers [52–54]. The studies of Zweig [55] and Perry [56] find that the prevalence of wealthy individuals in rural China is usually associated with communal envy, and mistrust. Yang and Zheng [57] compare the difference in the structure of social trust between urban and rural societies in China and find that social trust in the rural area is higher than that of city residents. They also find that with widening gap in economic development between urban and rural areas, city residents face deteriorating trust systems and values. Similarly, Hutchison and Xu [58] explore the determinants of political trust in China and find that provinces with high levels of inequality and openness are less trusting towards government. Using a large-scale survey for rural-urban migrants, Niu and Zhao [59] find that participating in religious-related activities has a positive impact on trust for both believers and non-believers.

## Data and methodology

### Data

Data on social trust and personal characteristics was obtained from the China Household Income Project (CHIP) 2013 survey. CHIP is used to measure and estimate the distribution of personal income in both rural and urban areas of the People's Republic of China. Data is available at: http://www.ciidbnu.org/chip. The survey data were collected through a series of questionnaire-based interview every four to five years and the existing CHIP surveys are CHIP1988, CHIP1995, CHIP2002, CHIP2007, and CHIP2013. The latest survey has not been yet publicly published. Therefore, we used the most recent publicly available survey data of 2013.

Regional population data and financial sector data came from the *China City Statistical Yearbook* (various issues). The religious institution data used in this paper comes from the Spatial Study of Chinese Religion and Society project (Center on Religion and Chinese Society, Purdue University. The “Spatial Study of Chinese Religion and Society” project was co-sponsored by the Center on Religion and Chinese Society at Purdue University, the China Data Center at the University of Michigan, and the Center for Global Studies at Purdue University Calumet. The project aimed at promoting research, teaching, learning, and training on the spatial study of religions and society in China). We matched the other data with the CHIP data according to location (city) and excluded those who lived outside the household in rural areas (e.g. those in schooling or in military service) in 2013. The total sample is 10,356 observations.

The social structure of rural China has changed along with the development of the economy. Industrialization, urbanization, agricultural modernization, population, and greater access to information have all contributed to this change [60–62]. It can be argued that these changes impinge on trust relations in rural China. According to the CHIP 2013 there are two kinds of social trust data, which are the trust of relatives or friends and the trust of others. We define trust of relatives or friends as *particular trust* (The respondents were asked to make single choice among “1. not trustworthy at all; 2. not very trustworthy; 3. moderate; 4. trustworthy; 5. very trustworthy; 6. unsure/no answer” when they answered the question “Would you say that your relatives or friends are trustworthy?”), and trust of others as *general trust* (The respondents were asked to make single choice among “1. not trustworthy at all; 2. not very trustworthy; 3. moderate; 4. trustworthy; 5. very trustworthy; 6. unsure/no answer” when they answered the question “Would you say that others (beside your relatives or friends) are trustworthy?”). Table 1 gives the distributions of the different levels of trust in our sample.

**Table 1:** Distributions of the different levels of trust.

Choice	*Particular trust* proportions (%)	*General trust* proportions (%)
1	2.135%	2.077%
2	2.859%	13.775%
3	26.468%	46.938%
4	47.324%	28.381%
5	19.561%	4.192%
6	1.652%	4.637%
Observations	10356	10356

Note: We excluded the choice 6 “unsure/no answer” from the regression sample. Hence, the sample size is reduced in regression analyses.

Table 1 gives the distributions of the different levels of trust in our sample.

The data in Table 1 shows that 47.3% and 19.6% of rural citizens think that their relatives or friends are trustworthy and very trustworthy, respectively, while only 28.4% and 4.2% of rural citizens think that others are trustworthy and very trustworthy, respectively. This gap provides the insight that the determinants of the two kinds of trust tend to be different. The choice 6 "unsure/no answer" has been excluded from both particular and general trust variables. The orders for both *particular trust* and *general trust* variables are not trustworthy at all = 1, not very trustworthy = 2, moderate = 3, trustworthy = 4, very trustworthy = 5.

### Empirical model

The dependent variable is the rate of trust levels, which are a self-rating system as follows; not trustworthy at all; not very trustworthy; moderate; trustworthy; very trustworthy. Following relevant previous studies, Ordered Logit model is used in our empirical study. Let Y_i_ be an ordered rating dependent variable that takes on the value of 1, 2, 3, 4, 5 depending on whether interviewee {i} chooses according to the self-rating system. Hence, for the self-reported trust feeling as the measure of the social trust level, the ordered logit model we used is set as follow:

$$Y_{i}=\alpha +\beta X_{i}+\varepsilon_{i}$$ (1)

where Y_i_ is a categorical variable that is observed as in Eq (2). X_i_ is the vector of independent variables, which includes measures of religion institutional density, financial development, and personal characteristics. *ε*_i_ is the error term.

Yi=1,Yi*<μ12,μ1<Yi*<μ23,μ2<Yi*<μ34,μ3<Yi*<μ45,Yi*>μ4 (2)

In Eq (2), μ_i_ stands for the i cut-point. Follow Long [63], the regression of ordered logit model (in terms of probability) is estimated as follows:

$$\mathrm{Pr}\left(Y_{i}>j\left|X\right.\right)=\frac{exp(X_{i}\beta^{'}-\mu_{i})}{1+exp(X_{i}\beta^{'}-\mu_{i})},\;j=1,2,3,4$$ (3)

where X_i_ stands for (k×1) vector of observed explanatory variables (k is the number of independent variables); β is a (k×1) vector of unknown parameters to be estimated. The parameters of the model are estimated by the method of maximum likelihood.

The independent variable for financialization is measured by the share of employees in the financial sector over total employees of the corresponding city, while Religion is measured by the number of religious institutions per 10000 people of the corresponding city. Other independent variables include the following personal characteristics; *Female* is a dummy variable that signifies if the interviewee is female (single = 1). *Age* is the interviewee’s age in 2013. *Health*, which is a self-evaluation dummy variable describing that if self-reporting current health condition is better than average of people of the same age. *Education* is a proxy for the formal education years of interviewee. The variable *income* stands for the total disposable income of the household. *Underloan* is a dummy variable that signifies if the interviewee was repaying a loan in the three years prior to the survey. *Communist* is a dummy variable that signifies if the interviewee was a CCP member. The variable *Cadre* tries to capture the personal identity difference between village/township cadre and others (*Cadre* = 1).

We excluded the choice 6 “unsure/no answer” from the regression sample (see Table 1). The details of the empirical variable definition and summary statistics are given in Table 2. Females constituted only a small proportion (8.9%). The formal education years are also heterogeneous. The highest formal education year is 20, while the lowest is only 1. The income of each household varies from 1,200 to 1,600,000 RMB, with a mean value of 44,797 RMB. The average percentage of employees in the finance sector over total employees is 3.4%. The average value of religious institutions per 10,000 people is 22.901; the highest and lowest value is 0.307 and 155.478, respectively.

**Table 2:** Definition and summary statistics of the variables.

Variables	Definition	Obs	Mean	S.D.	Min	Max
**Dependent variables**						
Particular trust	not trustworthy at all = 1, not very trustworthy = 2, moderate = 3, trustworthy = 4, very trustworthy = 5	10185	3.806	0.861	1	5
General trust	not trustworthy at all = 1, not very trustworthy = 2, moderate = 3, trustworthy = 4, very trustworthy = 5	9877	3.198	0.817	1	5
**Independent variables**						
*Finance*	Employees in finance sector over total employees of the corresponding city	9766	3.394	1.246	1.500	7.307
(%)						
*Religion*^a^	Religious institutions per 10000 people of the corresponding city	9766	0.229	0.299	0.003	1.554
*Female*	Female = 1, male = 0	10037	0.089	0.284	0	1
*Age*	Age in 2013	10037	49.835	11.521	18	87
*Health*	Thinking current health condition is better than average of people of the same age = 1, otherwise = 0	10037	0.667	0.471	0	1
*Education*	Formal education years	9974	7.176	2.689	1	20
*Income*	Total disposable income of the household	9978	45654	44797	1200	1600000
(RMB Yuan)						
*Underloan*	under borrowing in the last three years before doing the survey = 1, otherwise = 0	10037	0.301	0.432	0	1
*Communist*	Communist party member = 1, otherwise = 0	10037	0.111	0.313	0	1
*Cadre*	Village/township Cadre^b^ = 1, otherwise = 0	10037	0.055	0.228	0	1

Note: ^a^ Religions include Christian, Catholic, Islamic, Buddhist, and Taoist.

^b^ Village/township cadre includes the village branch secretary, director of the village committee, other village cadre, section-level township cadre and other township cadre.

### Empirical results

All of the data in the regression are Winsorised at the 1^st^ and 99^th^ percentiles (VIF results reveal that there is no multicollinearity among variables. The findings can be provided upon request). The results for the determinants of social trust in rural China are shown in Table 3.

**Table 3:** The determinants of social trust in rural China, ordered logistic regression.

	Particular trust			General trust		
	(1)	(2)	(3)	(4)	(5)	(6)
**Financial Development**						
*Finance*	-0.006**	-0.005**	-0.005**	-0.001	-0.001	
	(-2.363)	(-2.033)	(-2.018)	(-0.862)	(-0.846)	
**Religion**						
*Religion*	0.007	0.002		0.016***	0.015***	0.015***
	(0.652)	(0.168)		(5.889)	(5.683)	(5.688)
**Personal Characteristics**						
*Female*		0.007			-0.005*	-0.005**
		(0.660)			(-1.899)	(-1.928)
*Age*		0.001***	0.001***		0.004***	0.004***
		(3.356)	(3.391)		(4.358)	(4.368)
*Health*		0.051***	0.051***		0.011***	0.011***
		(7.422)	(7.402)		(6.421)	(6.425)
*Education*		0.003***	0.003***		0.001**	0.001**
		(2.581)	(2.563)		(2.468)	(2.453)
*Ln(Income)*		0.007*	0.007*		-0.003***	-0.003***
		(1.749)	(1.744)		(-3.077)	(-3.065)
*Underloan*		0.074***	0.074***		0.007***	0.007***
		(10.160)	(10.183)		(3.840)	(3.874)
*Communist*		-0.002			-0.000	
		(-0.023)			(-0.089)	
*Cadre*		0.044***	0.044***		0.010**	0.011***
		(2.822)	(3.063)		(2.379)	(2.576)
Pseudo R^2^	0.004	0.085	0.085	0.017	0.055	0.054
LR	16.08***	190.35***	189.89***	37.71***	117.83***	117.11***
Observations	9766	9541	9610	9766	9541	9672

Note: Average marginal effects are reported in the table (Particular trust & General trust = 5); Z statistics in parentheses.

* significant at 10%;

** significant at 5%;

*** significant at 1%.

### Benchmark model regression

It is notable that the results in regressions (1), (2) and (3) show that *Finance* has a negative relationship with *particular trust*. It is generally accepted that formal financial institutions are associated with fast growth and better resource allocation. An increase in the number of employees in the finance sector indicates an increase in financial activities and financial development, which enables access funds. The development of the finance sector in China has created greater access to formal loan channels in the rural areas, which acts as a substitute to informal loan from relatives and friends. This result is like Turvey and Kong [64], who argue that there is a positive relationship between formal loans and mistrust in rural China.

In regression (4)—(6), *Religion* has a positive relationship with general trust but insignificant relationship with particular trust, which suggests that religion is a source of trust for other people other than friends and relatives. A strong religious environment enhances trust. In addition, religion motivates altruism and service, while financialization encourages self-service and profit-taking (The unreported results show that Buddhism and Christianity have a positive and significant impact on particular trust, and Buddhism and Taoism have positive and significant impact on general trust. The other religions have no significant impact. Moreover, the unreported joint-effect analysis of financialization with different religions suggests positive and significant impact on particular trust. The only exception is Finance*Islam. While Finance*Islam has a negative and significant impact, Finance*Christianity has a positive and significant impact on general trust. A possible explanation for this differential result is that financial development in China has followed along the lines of conventional banking and interest payments which is contrary to Sharia law. Increased financialization may reduce general trust in areas of Islamic concentration. The results are available upon the request. We thank the anonymous referee for pointing out this).

As to the personal characteristics, the variable *Female* has a negative relationship with general trust, which suggests that women have higher likelihood to be suspicious of outsiders. The empirical result shows that *Age* has a positive relationship with both kinds of trust, which supports the notion that personal social status and experience increases with age, making people more trustworthy (We used Age^2^ in the regression but the result was insignificant. Therefore, we decided to use Age variable as it has high level of significance). The dummy variable *Health* is significant, highlighting that people in good health tend to be more trusting. This result is consistent with Alesina and Ferrara [65] and Feng et al. [66]. *Education* has a significant impact on *particular trust* at the 10% level, which gives a hint that more educated people tend to have more particular trust. Higher education level is typically associated with greater respect from friends and relatives, which may have positive effect on the attitude towards *particular trust*.

The variable *Income* has a positive and significant relationship with the probability of *particular trust*. This result is also consistent with Fehr’s [67] study. People with a higher income are usually considered to be more capable to deal with misplaced trust. However, income has a significant negative effect on general trust.

Table 3 also shows that *Underloan* has a positive relationship with both dependent variables, which implies that successful borrowing is a source of trust in general. Furthermore, the variable *Communist*, which stands for CCP membership, has an insignificant effect both on *particular trust* and *general trust*. The results suggest that party membership has no significant effect on social trust in rural China. This is reflective of the fact of lower relative CCP membership in rural areas. More interestingly, *Cadre* has a positive impact on the probability of social trust. Village and township cadres are usually composed of influential people with power and high social status in the local rural areas.

### Joint-effect analysis of financial development and religion

Table 4 reports the joint-effect analysis of financialization and religion on social trust. In regression (1), (2) and (3), the joint variable Finance*Religion has a positive relationship with *particular trust* when the marginal effect of *Finance* is still negative. The interaction impact of Religion is to help mitigate the negative effect from financialization on particular trust, which is a finding confirmed by Cao et al. [7] regarding religion and informal finance. It can be argued that financialization destroys the traditional relatives and friends trust based on lending and borrowing in rural area, while religiosity acts to attenuate the negative effect of financial development. However, the joint variable Finance*Religion is insignificant to general trust in regression (4), (5), (6), which indicates that the joint effect only exists on trust of relatives and friends, but Religion has an independent and positive association with *general trust*.

**Table 4:** Joint-effect analysis of financial development and religion on social trust.

	Particular trust			General trust		
	(1)	(2)	(3)	(4)	(5)	(6)
**Financial Development**						
*Finance*	-0.021***	-0.020**	-0.020**	-0.002	-0.002	-0.002
	(-6.432)	(-6.000)	(-5.974)	(-1.549)	(-1.428)	(-1.429)
**Religion**						
*Religion*	-0.135***	-0.139***	-0.139***	0.011***	0.012***	0.012***
	(-5.997)	(-6.047)	(-6.039)	(2.850)	(3.131)	(3.131)
**Joint-effect**						
Finance*Religion	0.048***	0.048***	0.047***	0.005	0.005	0.005
	(7.032)	(6.857)	(6.840)	(0.202)	(0.211)	(0.211)
**Personal Characteristics**						
*Female*		0.009			-0.005*	-0.005*
		(0.818)			(-1.803)	(-1.802)
*Age*		0.001***	0.001***		0.001***	0.001***
		(3.068)	(3.107)		(4.266)	(4.309)
*Health*		0.051***	0.050***		0.011***	0.011***
		(7.386)	(7.349)		(6.386)	(6.386)
*Education*		0.004***	0.003***		0.001**	0.001***
		(2.643)	(2.624)		(2.539)	(2.562)
*Ln(Income)*		0.006*	0.006*		-0.003***	-0.003***
		(1.756)	(1.759)		(-3.169)	(-3.171)
*Underloan*		0.073***	0.073***		0.007***	0.007***
		(10.087)	(10.091)		(3.803)	(3.803)
*Communist*		-0.001			0.000	
		(-0.011)			(-0.049)	
*Cadre*		0.046***	0.047***		0.010**	0.010***
		(2.922)	(3.183)		(2.435)	(2.638)
Pseudo R^2^	0.024	0.107	0.106	0.021	0.059	0.059
LR	55.68***	237.49***	236.82***	45.84***	127.64***	127.64***
Observations	9766	9541	9541	9766	9541	9541

Note: Average marginal effects are reported in the table (Particular trust & General trust = 5); Z statistics in parentheses.

* significant at 10%;

** significant at 5%;

*** significant at 1%.

### Joint-effect analysis of financial development and other variables

In this section, we check on the joint effect of financial development and other variables, in order to evaluate the effect of financialization on people's attitudes towards trust. The regression results are given in Table 5.

**Table 5:** Joint-effect analysis of financialization and personal characteristics on social trust.

	Particular Trust				General Trust			
	(1)	(2)	(3)	(4)	(5)	(6)	(7)	(8)
**Finance development**								
*Finance*	-0.005***	-0.006***	-0.004*	-0.005**	0.001	0.005	-0.000	-0.001
	(-2.688)	(-3.069)	(-1.866)	(-2.178)	(0.851)	(0.924)	(-0.445)	(-0.795)
**Religion**								
*Religion*	0.001	0.002	0.002	0.002	0.015***	0.015***	0.015***	0.015***
	(0.138)	(0.142)	(0.167)	(0.161)	(5.671)	(5.647)	(5.665)	(5.676)
**Personal Characteristics**								
*Female*	0.007	0.001	0.007	0.007	-0.005*	-0.005*	-0.005*	-0.005*
	(0.609)	(0.125)	(0.658)	(0.658)	(-1.959)	(-1.909)	(-1.892)	(-1.899)
*Age*	0.001***	0.001**	0.001***	0.001***	0.001***	0.001***	0.001***	0.001***
	(3.349)	(3.047)	(3.357)	(3.356)	(4.350)	(4.406)	(4.346)	(4.357)
*Health*	0.078***	0.049***	0.051***	0.051***	0.018***	0.011***	0.011***	0.011***
	(4.309)	(6.968)	(7.424)	(7.422)	(3.904)	(6.379)	(6.400)	(6.420)
*Education*	0.003**	0.013***	0.003**	0.003**	0.001***	0.004***	0.001**	0.001**
	(2.550)	(4.312)	(2.582)	(2.579)	(2.440)	(4.170)	(2.473)	(2.467)
*Ln(Income)*	0.007*	0.007*	0.007*	0.007*	-0.003***	-0.003***	-0.003***	-0.003***
	(1.774)	(1.775)	(1.749)	(1.746)	(-3.057)	(-3.065)	(-3.082)	(-3.078)
*Underloan*	0.074***	0.072***	0.074***	0.074***	0.007***	0.007***	0.007***	0.007***
	(10.167)	(9.719)	(10.162)	(10.159)	(3.857)	(3.802)	(3.838)	(3.839)
*Communist*	`-0.000	-0.002	-0.006	-0.000	-0.000	-0.000	0.007	-0.000
	(-0.043)	(-0.201)	(-0.211)	(-0.016)	(-0.115)	(-0.054)	(0.877)	(-0.088)
*Cadre*	0.044***	0.047***	0.044***	0.041***	0.010***	0.010***	0.010**	0.011**
	(2.825)	(2.906)	(2.826)	(2.487)	(2.377)	(2.321)	(2.356)	(2.428)
**Joint-Variables**								
Finance*Health	-0.018				-0.002			
	(-0.883)				(-1.543)			
Finance* Education		0.028***				-0.001***		
		(4.688)				(-3.473)		
Finance*			0.002				-0.002	
*Communist*			(0.220)				(-0.993)	
Finance*Cadre				-0.003				-0.000
				(-0.305)				(-0.115)
Pseudo R^2^	0.089	0.088	0.085	0.085	0.056	0.056	0.055	0.055
LR	193.21***	189.47***	190.40***	190.45***	120.53***	119.89***	118.82***	117.85***
Observations	9541	9541	9541	9541	9541	9541	9541	9541

Note: Average marginal effects are reported in the table (Particular trust = 5); Z statistics in parentheses.

* significant at 10%;

** significant at 5%;

*** significant at 1%.

Table 5 reports the joint effect of financialization (*Finance*) and other variables on *particular trust*. In regression (2), the effect of Finance*education on *particular trust* is significantly positive at 1% level. This result suggests that increasing the level of education can mitigate the negative effect from financial development on *particular trust*. In regression (6), the joint variable Finance*education has a negative effect at the 10% significant level on general trust, which provides evidence that higher educated people in financially developed areas tend to trust strangers less. It is notable that both the effects of Finance*Communist and Finance*Cadre have no significant relationship with the dependent variables, which suggests the personal political characterises cannot enhance or eliminate the influence of financialization.

### Joint effect analysis of religion and other variables

In this section, we have a further check on the joint effect of religion and other variables, in order to find out how religion changes attitudes towards trust. The regression results are given in Table 6.

**Table 6:** Joint effect analysis of religion and personal characteristics on social trust.

	Particular Trust				General Trust			
	(1)	(2)	(3)	(4)	(5)	(6)	(7)	(8)
**Finance development**								
Finance	-0.005*	-0.005*	-0.005*	-0.005**	-0.001	-0.001	-0.001	-0.001
	(-1.895)	(-1.927)	(-1.933)	(-1.978)	(-0.835)	(-1.241)	(-0.839)	(-0.806)
**Religion**								
Religion	0.053***	-0.041	0.002	0.003	0.001	-0.010	0.015***	0.014***
	(-2.674)	(-1.509)	(0.199)	(0.325)	(0.176)	(-0.890)	(5.238)	(5.221)
**Personal Characteristics**								
Female	0.007	0.007	0.007	0.008	0.005*	-0.006**	-0.005*	-0.005*
	(0.653)	(0.619)	(0.660)	(0.664)	(-1.884)	(-2.089)	(-1.898)	(-1.903)
Age	0.001***	0.001**	0.001***	0.001***	0.001***	0.001***	0.001***	0.001***
	(3.433)	(3.275)	(3.357)	(3.360)	(4.448)	(4.097)	(4.355)	(4.355)
Health	0.035***	0.051***	0.051***	0.051***	0.007***	0.011***	0.011***	0.011***
	(4.101)	(7.418)	(7.423)	(7.427)	(3.168)	(6.264)	(6.419)	(6.414)
Education	0.003***	0.002**	0.003**	0.003***	0.001**	-0.000	0.001**	0.001**
	(2.616)	(2.166)	(2.583)	(2.596)	(2.518)	(-0.212)	(2.462)	(2.450)
Ln(Income)	0.007*	0.007*	0.007*	0.007*	-0.003***	-0.004***	-0.003***	-0.003***
	(1.849)	(1.799)	(1.747)	(1.749)	(-2.965)	(-3.384)	(-3.072)	(-3.077)
Underloan	0.074***	0.074***	0.074***	0.074***	0.007***	0.006***	0.007***	0.007***
	(10.172)	(10.131)	(10.157)	(10.168)	(3.841)	(3.507)	(3.818)	(3.818)
Communist	-0.001	-0.000	0.001	-0.000	-0.000	0.000	-0.001	-0.000
	(-0.056)	(-0.013)	(0.050)	(-0.017)	(-0.107)	(0.034)	(-0.285)	(-0.102)
Cadre	0.044***	0.044***	0.044***	0.052***	0.010**	0.011**	0.010***	0.010***
	(2.795)	(2.799)	(2.824)	(2.726)	(2.354)	(2.533)	(2.373)	(2.326)
**Joint-Variables**								
Religion*Health	0.001***				0.002***			
	(3.233)				(3.460)			
Religion* Education		0.000*				0.001**		
		(1.702)				(2.205)		
Religion*			-0.000				0.003	
Communist			(-0.126)				(0.385)	
Religion*Cadre				-0.000				0.000
				(-0.693)				(1.218)
Pseudo R^2^	0.090	0.087	0.085	0.082	0.060	0.058	0.055	0.055
LR	200.77***	193.25***	190.37***	177.10***	129.79***	120.30***	117.98***	119.32***
Observations	9541	9541	9541	9541	9541	9541	9541	9541

Note: Average marginal effects are reported in the table (Particular trust = 5); Z statistics in parentheses.

* significant at 10%;

** significant at 5%;

*** significant at 1%.

Table 6 reports the joint effect of religion and other variables on particular trust. In regression (1), the joint variable Religion*Health has a positive relationship with the dependent variable. Being healthy keeps people in good mood, which is an important motivation for trust [68]. Hence, religious power can strengthen the effect of health on particular trust. In regression (2), the effect of the joint variable Religion*Education on particular trust is significantly positive at the 10% level. A positive relationship between education and trust in religion in China has also been found in Xie et al. [69], which support our finding that higher educated people in religiously prosperity areas tend to trust their friends and relatives more. As to general trust, in regressions (5) and (6), the joint variables Religion*Health and Religion*Education has a significant positive effect. Given that *Religion* has a positive relationship with trust level, it is notable that the joint variable Religion*Communist has no significant effect on general trust, which again support the notion that the trust behaviour of communist party members in rural China cannot be effected by religious power.

### Further check on income inequality

As suggested by Ward et al. [70] that low income leads to low trust, we conduct a sub-income analysis to explore if there are any differences in the determinants of social trust between high income and low-income groups (According to the regional average rural income data from the China Rural Statistical Yearbook (2014), we divide our sample into high income (above average) and low income (blow average) sub-groups). The results are shown in Table 7.

**Table 7:** The determinants of social trust in the rural China-sub sample analysis.

	Particular trust				General trust			
	High income	Low income	High income	Low income	High income	Low income	High income	Low income
**Financial Development**								
*Finance*	0.001	-0.013***	-0.015	-0.027***	0.001	-0.003**	-0.001	-0.005***
	(0.501)	(-3.592)	(-1.354)	(-5.364)	(0.947)	(-2.542)	(-0.680)	(-3.566)
**Religion**								
*Religion*	0.003	0.007	-0.150***	-0.121***	0.013***	0.019***	0.009***	0.004**
	(-0.011)	(0.416)	(-4.900)	(-3.444)	(4.300)	(4.117)	(3.087)	(2.509)
**Joint effect**								
Religion*Finance			0.050***	0.045***			0.000	-0.000
			(5.496)	(4.054)			(0.185)	(-0.368)
**Personal Characteristics**								
*Female*	0.010	-0.013	0.009	0.017	-0.003	-0.008**	-0.002	-0.007**
	(0.564)	(0.716)	(0.614)	(0.921)	(-0.816)	(-2.480)	(-0.774)	(-2.353)
*Age*	0.058***	0.001	0.001***	0.001	0.003***	0.001**	0.003***	0.001**
	(2.963)	(1.503)	(2.816)	(1.270)	(3.792)	(2.135)	(3.772)	(2.005)
*Health*	0.059***	0.038***	0.059***	0.037***	0.013***	0.007**	0.013***	0.007**
	(6.642)	(3.644)	(6.533)	(3.597)	(6.482)	(2.276)	(6.465)	(2.231)
*Education*	0.015**	0.002	0.004**	0.002	0.001*	0.001	0.001**	0.001
	(2.539)	(1.019)	(2.490)	(1.161)	(2.140)	(1.252)	(2.151)	(1.364)
*Ln(Income)*	0.016**	0.006	0.019***	0.006	0.001	-0.003**	0.001	-0.003**
	(2.538)	(0.885)	(2.560)	(0.875)	(0.773)	(-2.322)	(0.775)	(-2.321)
*Underloan*	0.070***	0.076***	0.071***	0.074***	0.006**	0.008**	0.006**	0.008**
	(7.438)	(6.763)	(7.449)	(6.670)	(2.803)	(2.400)	(2.791)	(2.362)
*Communist*	-0.001	0.002	-0.004	0.004	-0.000	-0.001	-0.000	-0.001
	(-0.223)	(0.143)	(-0.261)	(0.241)	(-0.061)	(-0.192)	(-0.067)	(-0.099)
*Cadre*	0.054**	0.041	0.049**	0.042	0.013**	0.005	0.013***	0.005
	(2.381)	(1.592)	(2.479)	(1.547)	(2.574)	(0.641)	(2.613)	(0.690)
Pseudo R^2^	0.092	0.084	0.126	0.101	0.075	0.046	0.078	0.053
LR	113.34***	80.82***	159.55***	97.35***	91.02***	43.35***	95.00***	49.68***
Observations	5060	4481	5060	4481	5060	4481	5060	4481

Note: Average marginal effects are reported in the table (Particular trust & General trust = 5); Z statistics in parentheses.

* significant at 10%;

** significant at 5%;

*** significant at 1%.

From Table 7, it can be seen that *Age*, *Health*, *Education*, *Underloan* and *Cadre* have a positive relationship with both kinds of trust in the high income group (income above regional average), while *Underloan* and *Health* are significantly positively linked to both kinds of trust in the low income group (i.e. income below the regional average). *Religion* has a significant positive impact on general trust for both high- and low-income groups, which supports the notion that religion creates greater irrespective of area. The findings show that religiosity attenuates the negative association between financialization and particular trust for low income group.

The key variable *Income* has differential effects on high- and low-income groups at different circles of trust. It has a positive effect on particular trust only in the high-income group and it has an insignificant effect on trust in the low-income group. However, *Income* has a negative effect on general trust to in the low-income group. The possible explanation for this phenomenon is that in poor areas, the envy by villagers towards rich neighbours results in general mistrust. This finding is consistent with Perry [56], in the study of Gansu province which is a typical low-income area in China. Given that income inequality is frequently observed in rural China [71], the results from the comparison between high- and low-income groups suggest that income inequality is a source of social mistrust in rural China. Furthermore, the results give a hint that the impact of income on trust has a threshold effect. When the average income in rural China rises above a certain level, social trust is unaffected. Our findings suggest that inequality and poverty result in a weaker trust environment in rural China.

The propensity to trust in low income groups decreases with financialization. Clearly, those on low income usually benefit less from financial development than those on high income. Consequently, financialization does not help low income people to be more trusting, which is a challenge if a harmonious society is a desired objective. As pointed out in Norris and Inglehart [72] religion persists most strongly among vulnerable populations, low income people in rural area tends to rely more on religion. The variable *Female* only has a negative effect on general trust in the low-income group and it has an insignificant effect on trust in the high-income group. In general, being healthy is a more important source for trust no matter in rich or poor area, while the power of religion can make peasant trust their neighbours and strangers more.

## Conclusion

We have used the CHIP survey data of 2013 to test the impact of financial development, religion, and Party membership, on social trust in rural China. In addition, this paper contributes to our understanding of the interactions of the above on social trust.

The triangular interaction of financialization, religiosity, and trust is a complex one and requires careful interpretation. The empirical findings demonstrate a negative and significant impact of financialization on *particular trust* and an insignificant impact on *general trust*. This implies that financialization lessens the need for informal loans from relatives and friends or from alternative financing channels. The intuition behind this argument is that inner trust facilitates co-operation and mutually supportive relations for informal sources of financing. When the share of the finance sector increases in the presence of an expanding financial system, individuals may choose to use formal tools to access funds.

When it comes to the role of religion, the findings lead us to the conclusion that it has a positive and significant relationship with *general trust* but generally insignificant relationship with *particular trust*. This finding suggests that religion fosters a more caring society. Since family and close friends are part of the inner circle of trust like religion, religion encourages their treatment of outsiders like insiders.

The results of the joint effect of financialization and religion on particular trust show that religion is helpful to mitigate the negative impact on particular trust; while the interaction effect of financialization and religion has insignificant relationship with general trust. On dividing the sample into high- and low-income groups, the interaction of financialization and religion in the regressions show similar results. However, financialization has a negative impact on both particular trust and general trust in low income group.

Moreover, we find that membership of the CCP has no effect on social trust in rural China. In other words, being a Party member does not provide a greater sense of trust than being a non-member. This finding can be reflective of the relatively lower proportion of CP membership in rural areas. However, being a member of the village or township cadre has a positive and significant impact on social trust. This result implies that high social status in local communities adds to social trust building and people tend to trust people with whom they interact regularly.

The empirical results show that social trust tends to increase as people age and with improved health. The analysis on a sub-sample of high income and low-income groups shows that income inequality has a negative and significant impact on social trust. High income people trust their relatives and friends but do not trust others. For low income people, financialization has a negative impact both on *particular trust* and general trust. Only good health and religion can mitigate the lack of trust caused by financialization.

Overall, the findings suggest a complex relationship between financialization, religion and membership of the Communist Party with social trust in China. These complex relations are often invisible determinants of social trust. There is a need for further study to address these issues. In particular, it would be interesting to see the impact of different religions on social trust in China with the most recent CHIP data.

## Supporting information

S1 File This file contains the raw data used in empirical studies. (ZIP)
